# Effects of corticosteroids and their combinations with hyaluronanon on the biochemical properties of porcine cartilage explants

**DOI:** 10.1186/s12917-015-0611-6

**Published:** 2015-12-04

**Authors:** Puntita Siengdee, Tiwaporn Radeerom, Similan Kuanoon, Thippaporn Euppayo, Waranee Pradit, Siriwadee Chomdej, Siriwan Ongchai, Korakot Nganvongpanit

**Affiliations:** Animal Bone and Joint Research Laboratory, Department of Veterinary Biosciences and Public Health, Faculty of Veterinary Medicine, Chiang Mai University, Chiang Mai, 50100 Thailand; Department of Biology, Faculty of Science, Chiang Mai University, Chiang Mai, 50200 Thailand; Thailand Excellence Center for Tissue Engineering and Stem Cells, Department of Biochemistry, and Center of Excellence for Innovation in Chemistry, Faculty of Medicine, Chiang Mai University, Chiang Mai, 50200 Thailand; Excellence Center in Osteology Research and Training Center, Chiang Mai University, Chiang Mai, 50200 Thailand

**Keywords:** Cartilage explants, Hyaluronic acid, Corticosteroids, Extracellular matrix, Osteoarthritis

## Abstract

**Background:**

Intra-articular injection of corticosteroids is used to treat the inflammatory pain of arthritis and osteoarthritis (OA), but our previous study found a deleterious effect of these steroids on chondrocyte cells. Hyaluronic acid (HA) injection has been suggested as a means to counteract negative side effects through replenishment of synovial fluid that can decrease pain in affected joints. To better understand the effects of corticosteroids on these processes, dexamethasone (Dex) and prednisolone (Pred) were administered to porcine cartilage explants at several concentrations with and without HA. We examined corticoid effects by determining sulfate-glycosaminoglycan (s-GAG) and uronic acid (UA) content of the explant media, and safranin-O staining of the cells. Analysis of lactate dehydrogenase (LDH) activity was conducted to assess cell cytotoxicity.

**Results:**

Dex treatment significantly reduced cellular cytotoxicity compared to the other treatment groups, especially with regards to the release of s-GAG, and protects against superficial proteoglycan damage. However, there was no difference between Pred and Dex, with and without HA, in the UA content remaining in porcine cartilage explants.

**Conclusions:**

The data suggest that combinations of Dex and Pred with HA did not have a significant effect on protection or enhancement of the articular cartilage matrix under the current conditions.

## Background

Osteoarthritis (OA) is a degenerative disease that affects the functional ability of movable joints. Progression of the disease not only leads to degeneration of the articular cartilage, but also affects the entire synovial joint complex, including the subchondral bone, joint capsule, ligaments, tendons, synovial membrane, and menisci [1]. A number of factors, such as environment, genetics, as well as changes in the metabolism of the joint tissues can affect the degeneration and progression of cartilage lesions [2, 3]. The predominant symptoms of OA are pain, stiffness, and functional limitation of the joint. Interventional treatments using pharmacological therapies that are concurrent with physiotherapy provide symptomatic relief and aids physical therapy effectiveness [4].

Intra-articular (IA) injections of corticosteroids are often used in relieving pain via inhibition of the accumulation of inflammatory cells and mediators [5, 6]. The most common anti-inflammatory corticosteroids in veterinary medicine for relief of pain are dexamethasone (Dex) and prednisolone (Pred) [7]. Both are effective against inflammatory diseases and act by inhibiting inflammatory agents, such as PGE2 [8] and interleukin-1β [9]. IA injections of Dex or Pred are recommended in OA therapy because of their proven effectiveness in controlling chronic inflammation and pain [8–10]. However, many studies have shown that long-term IA corticosteroid injections have adverse effects, including enhanced progressive damage and increased risk of joint destruction. These events are brought on through decreased chondrocyte proliferation, induced cell apoptosis, and extracellular-matrix formation [11, 12], together with cartilage degeneration [12–14].

Hyaluronan (HA), or hyaluronic acid, is composed of repeating sequences of disaccharides, glucuronic acid, and N-acetylglucosamine. Intra-articular injections of HA are given to restore the viscoelasticity of the synovial fluid by inducing synoviocytes and chondrocytes to synthesize natural HA and inhibit the degradation of endogenous HA [15–18]. This is well documented in human and animal studies: injections have been shown to decrease pain and improve functional outcomes in OA as well as decrease inflammation in the joints [15–20]. However, the combined effect of steroids and local HA on chondrocytes has not been studied. Thus, the present study was conducted to determine if the simultaneous use of HA and corticosteroids (Dex and Pred) could reduce the adverse effects of corticosteroids on the biochemical properties of porcine cartilage explants in vitro.

## Methods

### Sample population

Normal porcine articular cartilage specimens (no osteoarthritis lesions) were prepared from the carpal and the tarsal joints of pigs that were ~20–24 weeks old (*n* =16), using an aseptic technique. The joints were received from a local slaughter house and processed within 6 h after slaughter, as described before [21]. The methods were carried out in accordance with the approved guidelines. The experimental protocol was approved (2014) by the Scientific and Ethics Committee, Faculty of Veterinary Medicine, Chiang Mai University, Chiang Mai Thailand.

### Reagents

Low molecular weight HA for IA injection (~500–730 kDa) was obtained from TRB Chemedica (Bangkok, Thailand). HA was diluted with Dulbecco’s modified Eagle’s medium (DMEM, Gibco Life Technologies) without serum and used for the experiment at the final concentration of 2.5 mg/ml. Three different concentrations (12.5 %, 25 %, and 50 %, respectively) of each of the drugs, Pred (L.B.S. Laboratory, Bangkok, Thailand) and Dex (T.P. Laboratories, Bangkok, Thailand), were established from the stock concentration of the drugs and diluted with serum-free DMEM (end concentrations: Pred at 3.125 mg/ml, 6.25 mg/ml, and 12.5 mg/ml, and Dex at 0.5 mg/ml, 1.0 mg/ml, and 2.0 mg/ml).

### Porcine cartilage explant cultures and experimental designs

#### Porcine cartilage explant cultures

The explants were prepared following the method of Chaiwongsa [21]. In brief, the joints were opened aseptically and pieces of cartilage were sliced from the condyles of the carpal and the tarsal joints; the average size of these pieces was ~7-8 mm long by ~1.5-2 mm deep and by ~2-4 mm wide. All of the explants were washed once with 70 % ethanol for 30 s, followed by washing three times with sterile phosphate buffered saline (PBS). Three pieces of the explants were placed into wells (~30–35 mg/well) of sterile 24-well culture plates. The cartilage disks were then incubated in 1000 μl serum-free DMEM until the start of the experiment, within 24 h. To investigate the effect of Dex and Pred combined with HA, the cartilage disks were cultured in serum-free DMEM (negative-control) that contained 200 units/ml of penicillin, 200 μg/ml of streptomycin, and 50 μg/ml of gentamicin. Treatment groups were inoculated with serum-free DMEM containing varying concentrations of Dex or Pred co-treated with or without HA to create 14 treatment groups in replication of three. Then, the cartilage disks were further incubated for 14 days as described below.

#### Experimental design

The experimental design of the porcine cartilage explant treatments is presented in Table 1. All cultures were maintained at 37 °C under 5 % CO_2_. The conditioned media were collected immediately before treatment (day 0) and 14 days after treatment. The cconditioned media and the cartilage explant disks were harvested and stored at −20 °C until further analysis. The cell cytotoxicity was measured based on the level of enzyme lactate dehydrogenase (LDH) in the conditioned media. The conditioned media and the cartilage explant disks were assayed for the release of biomolecules through cartilage matrix degeneration assay. Additionally, the cartilage explant disks were examined for the purpose of histopathology observation.

**Table 1:** Experimental group in this study

Control groups			
Name	Condition		
Control	DMEM without serum		
HA	HA 2.5 mg/ml		
**Treatment groups**			
Pred		Dex	
Name	Condition	Name	Condition
Pred50	Pred 50 %	Dex50	Dex 50 %
P50 + HA	Pred 50 % with HA 2.5 mg	D50 + HA	Dex 50 % with HA 2.5 mg
Pred25	Pred 25 %	Dex25	Dex 25 %
P25 + HA	Pred 25 % with HA 2.5 mg	D25 + HA	Dex 25 % with HA 2.5 mg
Pred12.5	Pred 12.5 %	Dex12.5	Dex 12.5 %
P12.5 + HA	Pred 12.5 % with HA 2.5 mg	D12.5 + HA	Dex 12.5 % with HA 2.5 mg

### Cytotoxicity assay

#### Lactate dehydrogenase (LDH) assay

The toxicity effect of all treatment conditions on the viability of chondrocytes within the explant disks was determined by colorimetric assay of LDH activity in the culture media [22]. The conditioned media of the cartilage explants disks were examined using CytoTox 96® Non-Radioactive Cytotoxicity Assay (Promega, USA) according to the manufacturer’s instructions and by comparing with the untreated media.

### Determination of cartilage matrix degradation

#### Sulfated glycosaminoglycan (s-GAG) content

The degradation of s-GAG was determined by measuring the s-GAG quantity in the culture media, which indicated biochemical changes in the articular cartilage. The S-GAG concentration was measured using a colorimetric dye (dimethylmethyleneblue [DMMB]) binding assay that was modified from a previous study [23]. Briefly, chondroitin 6-sulfate from shark cartilage (CS-C) (Sigma-Aldrich, USA) was used as the standard, with concentration values ranging from 0 μg/ml to 100 μg/ml. CS-C standards or diluted media samples (50 μl) were pipetted into 96-well plates. Then, 200 μl of the DMMB dye solution was immediately added to each well. Serum-free media was used as the blank. The absorbance of the solution was immediately measured at 540 nm using a microplate reader spectrophotometer and concentrations of s-GAG calculated [24].

#### Quantification of uronic acids (UA)

UA is a component of hyaluronic acid and chondroitin sulfate in the cartilage matrix, and was measured using a colorimetric assay: the carbazole analysis was modified according to the method discussed by Cesaretti [25]. Treated cartilage disks were digested with 200 μl of papain, 2 units, at 60 °C for ~48 h, and papain cartilage-digested media was diluted with distilled water at 1:5. Glucuronolactone was used as the standard reagent. A volume of 50 μl of diluted digested sample, or standard, was added with 300 μl of borate/sulfuric acid reagent (0.025 M Na_2_B_4_O_7_ in conc. sulfuric acid). Thereafter, the samples were heated at 100 °C for 15 min. When the sample cooled down to room temperature, carbazole in ethanol was added. After heating at 100 °C for ~15 min and cooling down to room temperature again, the quantity of uronic acid in the sample was measured at 540 nm, and the uronic acid content was estimated from the following calculation:

$$ \mathrm{U}\mathrm{A}\ \mathrm{content}\ \left(\%\upmu \mathrm{g}/\mathrm{mg}\ \mathrm{of}\ \mathrm{dry}\ \mathrm{tissue}\right)=100\times \left(\mathrm{U}\mathrm{A}\ \mathrm{Day}\ 14\ \mathrm{treated}\ \mathrm{sample}/\mathrm{weight}\ \mathrm{of}\ \mathrm{dry}\ \mathrm{tissue}\right) $$

### Histopathology study

The cartilage disks were fixed in 10 % neutral buffer formalin and subsequently embedded in paraffin wax. Cartilage sections of approximately 6 μm were prepared. The sections of each sample were stained with common haemotoxylin and eosin (H&E) and safranin-O, the latter of which is used to determine the proteoglycan content. Then, the cartilage sections were imaged by a Biocom microscope using an AxioCam camera (Carl Zeiss, Germany). The H&E stained samples were graded and given scores for the cartilage structure, which was followed by chondrocyte pathology according to the recommended protocol of the OARSI histopathology initiative [26]. The intensity of safranin-O staining of cartilage sections was used to quantify the proteoglycan content [27]. These were done by processing and calculating the stained image using ImageJ [28] 1.47 morphometric computer software (Wayne Rasband, National Institute of Health, USA).

### Statistical analysis

Values were expressed as mean ± standard deviation (S.D.) and the data analyzed using SPSS program version 17 for the Windows software package. Significant differences among treatment groups, except for histopathology data, were determined using one-way analysis of variance (ANOVA) at a significance level of *p* < 0.05. Differences in grades of histopathology staining were determined using the Kruskal–Wallis test.

## Results

### Effect of steroids in combination with HA on cell cytotoxicity in cartilage explant culture systems

**Fig. 1:**
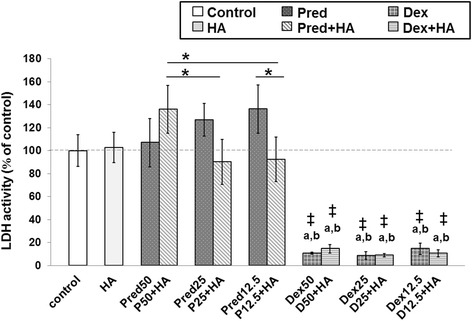
The effect of Pred and Dex in combination with HA on cell cytotoxicity. The LDH content of the cartilage explant treatments was calculated and compared with the control group. After the 14-day treatment period, the culture media were analyzed for LDH release, as previously described. The results are expressed as a percentage relative to the control group. The white bar denotes the control; the light gray bars denote the HA-treated or the co-treated groups; and the dark gray bars denote the steroid-treated groups. The data are expressed as the mean ± SD (*n* = 3). Significant differences are considered to exist when p < 0.05: “a” stands for significant differences compared with the control group; “b” denotes significant differences compared with HA; dagger (‡) stands for significant differences compared with all Pred-treated groups; and asterisk (*) denotes significant differences between the treatment groups

Cytotoxicity tests were conducted to evaluate the effect of steroids on porcine chondrocytes in cartilage explant culture systems. After treatment of the cartilage disks with steroids with and without HA, an analysis of LDH content was carried out using an LDH activity assay. The release of LDH into the culture medium from the damaged cells (cell loss of integrity) indicated an inclination toward cell death. The LDH content increased gradually in the Pred-treated groups from 50 %, 25 %, and 12.5 % concentrations (Fig. 1). At the same time, a significant inverse trend (decrease) was observed for the combination of Pred and HA, starting from P50 + HA, compared with the Pred-treated groups, with a marked decrease in P25 + HA and P12.5 + HA, compared with Pred50 + HA (*p*<0.05), which had slightly lower LDH content than the control and the HA groups (*p*>0.05). For Pred 12.5, the HA co-treatment reduced the cytotoxicity of the chondrocytes in the system at *p*<0.05 (from 136.28 % to 92.40 %, relative to the control group). However, no difference in LDH content between all of the Pred-treated groups and the control/HA treated group was found (*p*>0.05). For the Dex-treated group, a significant difference was found between both the Dex-treated and the Dex + HA-treated groups when compared with the control, the HA group, and all Pred-treated groups (*p*<0.05). Nevertheless, there were no significant differences found between the Dex-treated groups and the Dex + HA-treated groups in LDH content (Fig. 1). Notably, the mean percentage of the LDH content was reduced (*p*<0.05) in all the Dex and the Dex + HA treatment groups (8.80–14.72) in comparison with the control group, which was set as 100.

### Effects of Pred and Dex in combination with HA on release of s-GAG

**Fig. 2:**
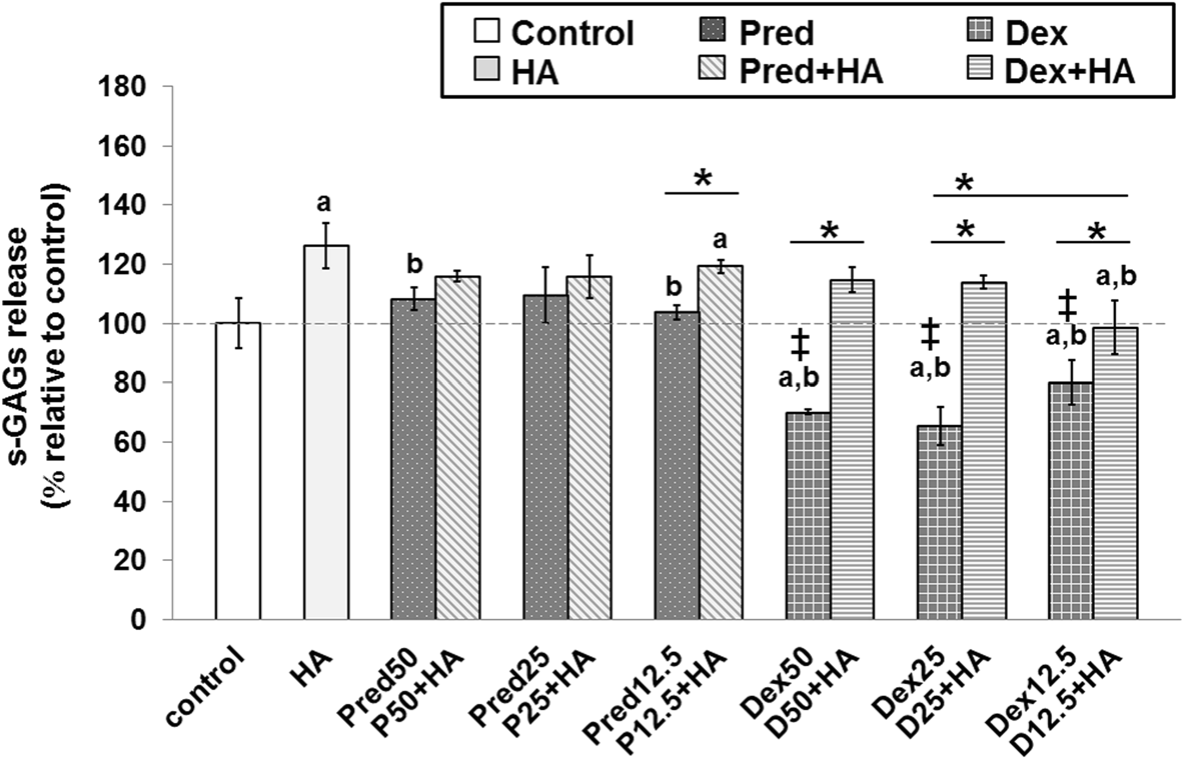
The effect of Pred and Dex in combination with HA on the release of s-GAGs. The percentage of the s-GAG content in the cartilage explant treatments was calculated and compared with that of the control group. After 14 days of treatment, the culture media were analyzed for the s-GAGs released into the media. The results are expressed as a percentage relative to the control. The white bar denotes the control; the light gray bars denote HA-treated or the co-treated groups; and the dark gray bars denote the steroid-treated groups. The data are expressed as the mean ± SD (*n* = 3). Significant differences are considered to exist when *p* < 0.05: “a” stands for significant differences compared with the control group; “b” denotes significant differences compared with the HA group; dagger (‡) stands for significant differences compared with all the Pred-treated groups; and asterisk (*) denotes significant differences between the treatment groups

The in vitro effects of HA in combination with Pred and Dex treatment on cartilage explant disks are shown in Fig. 2. At the end of the experiment, the culture media was evaluated for s-GAG which is the carbohydrate part of the aggrecan molecule and is a marker of cartilage degradation. The result showed a significant increase in the levels of s-GAG in the culture media of all HA and HA combination groups in comparison with the control. All of the Pred-treated groups at doses of 50 %, 25 %, and 12.5 % increased the release of s-GAG compared with the untreated group, but still increased the s-GAG level when compared with the single HA-treated group (*p*<0.05). Dex treatment protected against the release of s-GAG into the culture media, compared with the control (*p*<0.05) and HA-treated groups. Surprisingly, at lower concentrations, Dex was more effective than Pred against the release of s-GAG (*p*<0.05).

### Pred and Dex increase UA content from cartilage tissues

**Fig. 3:**
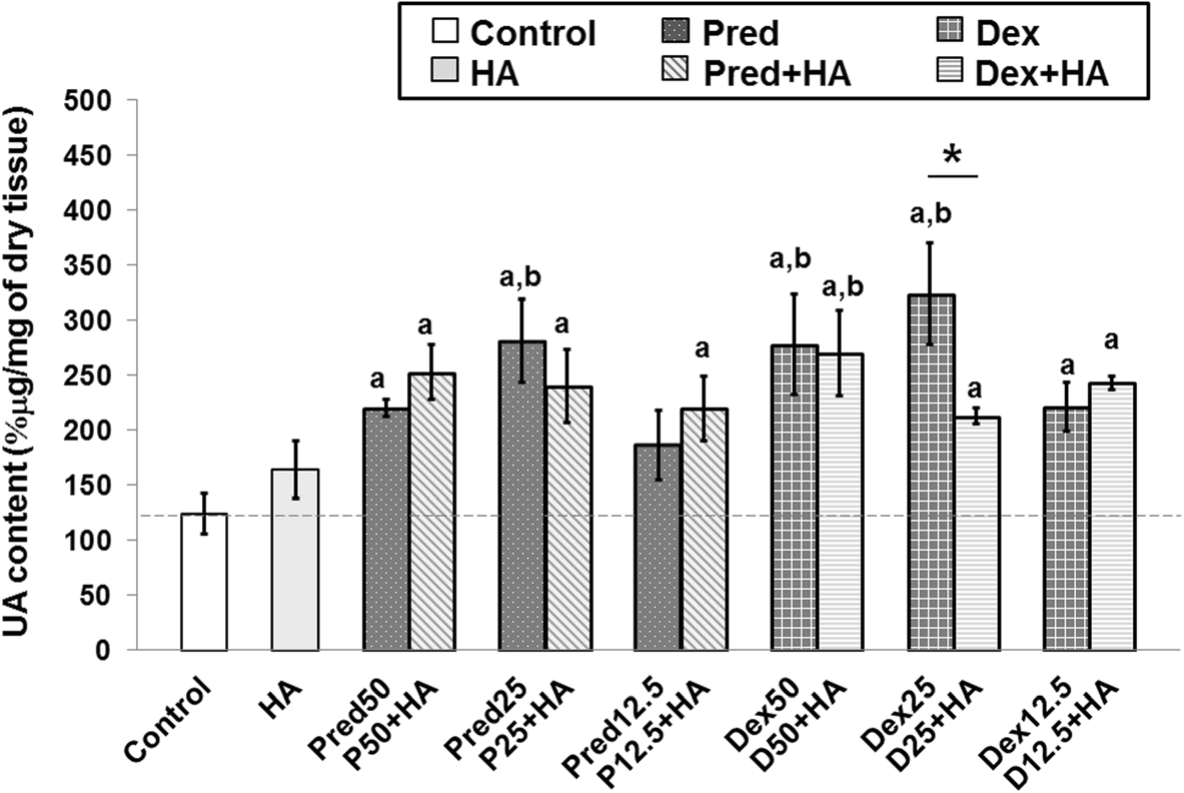
The percentage of uronic acid (UA) content patterns of steroids in combination with the HA treatment. The UA content of the cartilage explant treatments was calculated and shown in % μg/mg of dry tissue. The white bar denotes the control group; the light gray bars denote the HA-treated or the co-treated groups; and the dark gray bars denote the steroid-treated groups. Error bar: standard deviation of each group (*n* = 3 for each group). Significant differences are considered to exist when *p* < 0.05:“a” stands for significant differences compared with the control group; “b” denotes significant differences compared with the HA group; and asterisk (*) denotes significant differences between the treatment groups

We next determined the UA content, which is a class of monosaccharide derivative monomers of the glycosaminoglycans of proteoglycans and HA, using the DMB assay and results are shown in Fig. 3. The percentages of UA content in most of the steroid-treated groups (except Pred12.5 and single HA-treated groups) were significantly higher when compared with those of the control and the HA-treated group (*p*<0.05). Comparing the control and the single HA-treated groups, a slight improvement in the UA content was observed in the HA treatment (*p*<0.05). Of these, the most effective working concentration was found to be the 25 % treatment in both Pred and Dex because it significantly increased the UA content in cartilage explant disks compared to the control and the other treatment concentrations (*p*<0.05). In this study, co-treatment of HA could not be attributed to UA preservation; in addition, the co-treatment of HA at Dex25 (Dex25 + HA) could not counteract the destructive effects of Dex nor significantly lower the content of UA in the explants when compared with the Dex25-treated group (*p*<0.05). Overall, comparing between the Pred and the Dex treatments, Dex appeared to be more effective in improving both the s-GAG release and the UA content that remained in the cartilage disks.

### Histological analysis

**Fig. 4:**
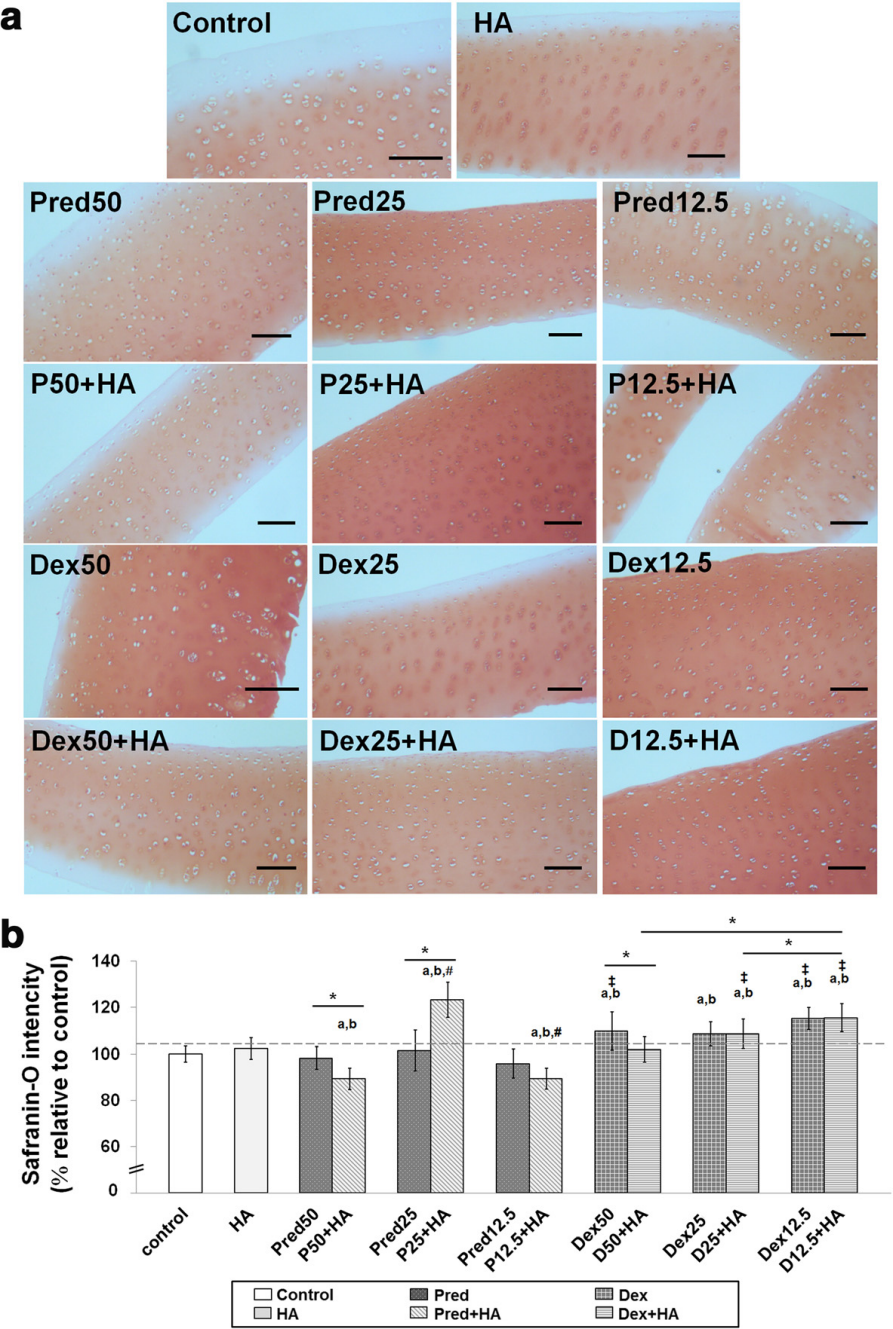
Histological staining with safranin-O (×100) of conditioned porcine cartilage disks. After 14 days of treatment with various concentrations of corticosteroids in combination with HA, the proteoglycans content in the cartilage tissue stains is indicated by safranin-O staining. The extent of the morphological change and the proteoglycans content were compared between all of the treatment groups. (**a**) Safranin-O staining representative of the results are shown start from upper row and left to right: control, HA, Pred50, Pred25, Pred12.5, P50 + HA, P25 + HA, P12.5 + HA, Dex50, Dex25, Dex12.5 and the last row D50 + HA, D25 + HA, D12.5 + HA, respectively. The Dex and the HA-treated groups performed rather efficiently, as indicated by the intense red hue, better than the other groups, including the control. The scale bars represent 100 μm. (**b**) An increase in the safranin-O intensity (relative to the control) in the steroid treatment groups is shown in the bar chart which is exhibited as the mean ± SD of the triplicate independent. Significant differences are considered to exist when *p* < 0.05: “a” stands for significant differences compared with the control group; “b” denotes significant differences compared with the HA group; dagger (‡) stands for significant differences compared with all the Pred-treated groups; hash (#) denotes significant differences compared with all the Dex-treated groups; and asterisk (*) stands for significant differences between the treatment groups

**Fig. 5:**
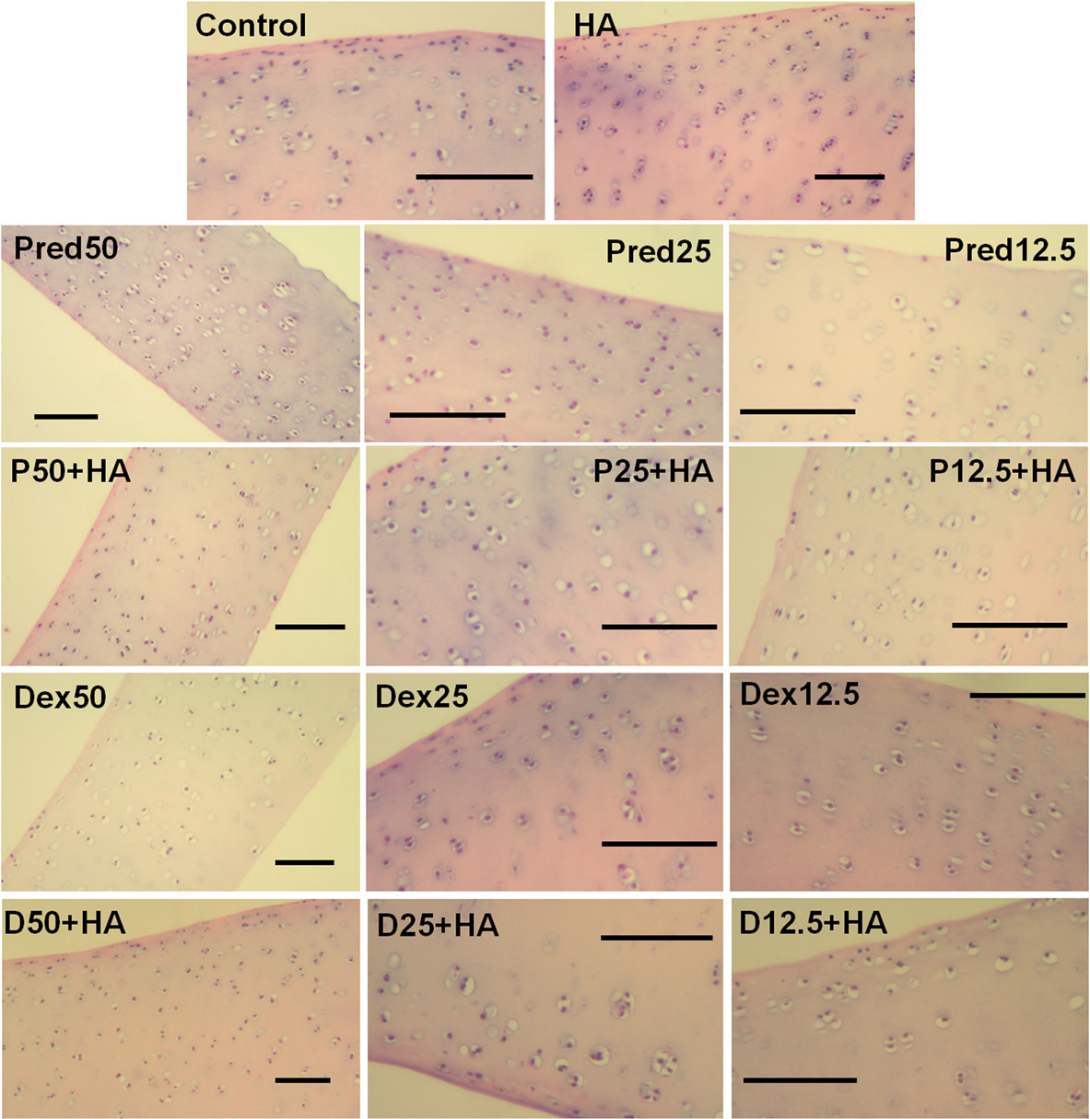
The histological section of porcine cartilage explants following 14 days of treatment with different and various concentrations of corticosteroids in combination with HA: H&E. The articular cartilage showing both the regular and the irregular cell clusters (H&E staining, 100×). The scale bars represent 100 μm

Results of histological analysis of cartilage explant-disks after 14 days of Pred and Dex treatment in combination with HA are shown in Fig. 4. Cartilage disk sections were stained with safranin-O to identify proteoglycan content. A significant increase in safranin-O staining was observed in the steroid-treated cartilage disks compared to controls, indicating that the treatments with Pred and Dex had enhanced the proteoglycan content in the matrix of the explant cultures or inhibited the cartilage destruction. We found that the superficial zone in nearly every treatment group, including the control and the HA groups, exhibited faded safranin-O intensity. Subsequently, the images were processed using the ImageJ analysis program, and plotted as a bar chart (Fig. 4b). The major increase in intensity was found in the co-treatment Pred25 + HA group, and was higher than that of the controls (*p* = 1.137 × 10^−12^). Moreover, the groups that underwent Dex treatment produced better results in proteoglycan content preservation when compared with the Pred-treated groups (*p* = 1.136 × 10-^12^ ,0.05 and 2.015 × 10^−08^ at 50 %, 25 %, and 12.5 % concentrations, respectively). Results of H&E staining to identify cell morphology, matrix structure, and composition in the treated explants are shown in Fig. 5. The morphology of the chondrocytes that were embedded in the treated cartilage explants was evaluated. Fibrillation on the articular surface and the chondrocyte characteristics, such as cell cluster formation and chondrocyte cloning and empty lacuna were considered. Nevertheless, the grading scores of the chondrocyte characteristics of the HA-treated group and those of the combination treatments with both Pred and Dex at 12.5 % and 25 % revealed slight differences (grade 1) in comparison with the other groups (normal, grade 0).

## Discussion

The pathogenesis of OA is caused primarily by an imbalance in joint metabolism; for example, when catabolism exceeds anabolism, which leads to degradation of the cartilage matrix [29]. Progressive cartilage loss makes the cartilage lose its normal shape, which leads to progressive painful and functional impairment [1]. Current therapeutic treatment options for OA are aimed at reducing pain, improving joint function, and limiting progressive cartilage loss, all of which are intended to maintain a good quality of life. However, medications used in the management of OA need to take into consideration possible side effects that bring about further deterioration of the articular cartilage. Traditionally, IA injections of corticosteroids have been used concurrently to provide short-term pain relief for patients with degenerative joints via inhibiting the accumulation of inflammatory cells and mediators, thereby subsequently decreasing swelling, diminishing pain, and improving motion [5, 6, 30]. In particular, IA corticosteroid injections for OA have proven effective for chronic pain management and preventing the progression of cartilage degeneration in the early stages of OA, and are recommended when signs of local inflammation arise [31, 32]. They are especially useful in patients who have not had success with NSAIDs or cannot use them because of gastrointestinal side effects. Nevertheless, derivatives of corticosteroids have been reported to have cytotoxic effects on various cell types, including chondrocyte cells. These side effects include inhibition of chondrocyte cell growth and induction of cell apoptosis [11–14], resulting in the loss of extra-cellular matrix formation and progressive degradation of the articular cartilage [12]. However, there are reports that corticosteroids do not directly cause chondrocyte death, but rather the effect is due to the presence of the preservative agent, benzalkonium chloride, in the injectable suspension [33]. Alternatively, supplementary HA injection has been recommended for OA for pain relief and improved joint function [32, 34, 35], although its effects on articular cartilage have not been clearly established [31, 36]. More recently, treatment strategies using corticosteroids in combination with HA injections have been shown to improve both pain and joint function, and are superior to treatment with HA alone [34, 37].

In our study, we used normal porcine cartilage explants as an in vitro model for examining the direct effects of the most commonly used corticosteroids, Dex and Pred, in addition to subsequent co-culturing with HA combinations on the articular cartilage. We compared the in vitro extracellular matrix molecules, cell cytotoxicity, and morphologic changes of the porcine articular cartilage explants after exposure to corticosteroid and HA. The experiment was designed for clinical application by avoiding external serum growth factor conditions that are associated with the cartilage extracellular matrix composition and cellularity changes [38] and excessive cell proliferation [39]. As a result, the culture medium was changed from supplementary 10 % to serum-free when the treatment was initiated, and the serum-free medium was used for the control group.

There are no reports of using these steroids in an in vitro cartilage explant model, so doses were chosen that represented two-fold serial dilutions of commercially available stock preparations. The concentration of HA was chosen based on a previous study of Euppayo [40]. The study duration of 14 days of porcine cartilage explant culture is the maximum duration recommended for cartilage graft storage, as mentioned in Pallante [41]. Based on the LDH assay results, cytotoxic effects were observed after Pred treatment at the highest concentration (50 %, or 2.0 mg/ml). In the combination treatment, however, inclusion of HA reduced the Pred cytotoxic effects and exhibited a protective effect when compared with the control and HA-only treated groups, while the Dex treatment groups dramatically decreased cell cytotoxicity in explant models when compared with others. Remarkably, this is inconsistent with the findings of previous studies. Dex has been found to induce cell death and suppress cell proliferation in cell types of many species, including chondrocytes and tendon cells, even at lower concentrations [11, 42, 43]. The results also contrasted with our preliminary study using a monolayer culture, where there was trend towards chondrocyte cell death in human articular cartilage in accordance with Dex concentration, whereas Pred treatment had little effect [44]. A determination of cartilage matrix degradation in both s-GAGs released and UA content that remained in the cartilage disks was carried out. Dex was found to be extremely effective—much more than Pred—against s-GAG loss and highly efficient in enhancing UA content in the cartilage explant disks compared with the control. Of these, the most effective concentration of Dex was found to be 25 %.

Safranin-O staining intensity is representative of the amount of proteoglycan remaining in the cartilage disks. In this context, the Dex50-treated groups were observed to have safranin-O dark stains, which refers to all of the proteoglycans remaining in the matrix, whereas at the superficial zone in the control, HA treatment and Pred-treatment groups, the safranin-O intensity was faded, implying the loss of some proteoglycan. Culture conditions in this study were similar to those in the study of Moo [45] in bovine articular cartilage. Scientific findings for the application of cartilage explants revealed that the proteoglycan content insignificantly decreased gradually over time in long-term culture until 17 days [45], and a variety of matrix content changes in the superficial zone was measured under various culture conditions [41, 46]. These occur together through peripheral collagen network degradation [45, 47]. Some changes were observed in the H&E histological results in groups of single/combination with HA treatments. Cartilage-treated specimens were observed to present the occurring of minor clusters of cell dispersing in the OA articular cartilage. The clusters arose due to the uniformity in the extracellular matrix of the cartilage, which is associated with some degree of cell death [48].

The results of cell cytotoxic effect and cartilage matrix degradation investigation were consistent in this study, but did not support our hypothesis that HA would be protective against the negative side effects of corticosteroid treatment. Rather HA combinations showed more destructive effects and did not cause significant enhancement of the articular cartilage matrix when exposed to corticosteroid alone and the control group, in particular, the Dex treatment. However, it was found that co-treatment of HA with Pred was successful in reducing the cytotoxic effects of this drug.

Currently, two molecular weights of HA, low (500–730 kDa) and high (6–7 million Da), are available, and each displays distinct pharmaceutical effects [35]. High molecular weight hyaluronic acid preparations are highly effective in relieving pain and have greater anti-inflammatory activity in vivo [35], while low molecular weight HA is similar to native hyaluronic acid. In particular, low molecular weight HA has shown impressive effects in many in vitro studies [49] in terms of activated proliferation and matrix synthesis [50], and reduced apoptosis of chondrocytes by inhibiting two specific receptors: anti-CD44 and anti-ICAM-1 [51]. Nevertheless, the effects of HA treatment are dependent on its molecular weight, concentration, and application duration [36, 52], in regards long-term pain relief [32, 34, 53]. The HA effects observed in this study are different from those observed in many previous clinical studies [32, 34, 53]; this could be because of the limited drug exposure duration in these culture systems, which resulted in limitations in the determination of the HA-treatment effects. There also could be differences due to in vitro vs. in vivo model systems, the latter of which involves more complex interactions between adjacent tissues of the joints. Last, it could be the result of low molecular weight of HA having more adverse effects, resulting in changes in the downstream signaling modification in the disease process, as discussed and reviewed elsewhere [35, 52, 54–56].

Overall, when a comparison is made between Pred and Dex treatments, Dex scores distinctively better in the action against s-GAG degradation and improvement in the UA content remaining in cartilage disks; it might also improve cell cytotoxicity in the culture condition. In combination with the findings of previous preliminary studies, it can be concluded that short-term Dex treatment is effective in suppressing the catabolic activities of pro-inflammatory cytokines by inhibiting the phospholipids, together with preventing proteoglycan degradation and restoring the matrix production [30, 46, 57, 58]. This is similar to observations in the culture systems of human and bovine bone marrow stromal cells [59]. Hence, it is speculated that besides its excellent anti-inflammatory effect, Dex may play a role in maintaining the mechanical functions as well as enhancing the matrix formation and the functional properties of articular cartilage.

Some limitations of this research must be considered. First, we were limited in the duration to which the explants were exposed to the drug treatment; therefore, the results might not reflect the complex effects in comparison with in vivo or clinical trials. Second, we utilized a single concentration of HA-treated combinations and chose to select the most commonly used glucocorticoids agents. In this regard, based on other reports and our previous preliminary data, different variations and combinations of HA treatment as well as the direct effects of Pred and Dex in chondrocytes should be subjected to further in-depth evaluation.

## Conclusions

These findings provide basic knowledge on the efficacy of selected corticosteroids to treat articular cartilage damage and prevent of the same via use of these drugs in combination; the consequences of the treatment point to the possibility for application of these mechanical effects in the clinical environment. Results from this study indicate that Dex is an effective drug that has rehabilitation possibilities in the treatment of OA and may be efficacious in protecting against the loss of glycosaminoglycan molecules without causing chondrocyte cell death. Additionally, HA combinations have not been completely elucidated and are still a matter of concern. We suggest that if this combined treatment cannot be avoided, then an appropriate treatment duration should be provided.

## Abbreviations

Dex: dexamethasone
DMEM: Dulbecco’s modified Eagle’s medium
DMMB: dimethylmethyleneblue
H&E: Haemotoxylin and Eosin
HA: Hyaluronic acid
IA: Intra-articular
LDH assay: Lactate dehydrogenase assay
NSAIDs: Nonsteroidal anti-inflammatory drugs
OA: Osteoarthritis
Pred: Prednisolone
s-GAG: Sulfate-glycosaminoglycan
UA: Uronic acid

## References

[1] Martel-Pelletier J, Boileau C, Pelletier J, Roughley P (2008). Cartilage in normal and osteoarthritis conditions. Best Pract Res Clin Rheumatol.

[2] Tat S, Pelletier J, Vergés J, Lajeunesse D, Montell E, Fahmi H (2007). Chondroitin and glucosamine sulfate in combination decrease the pro-resorptive properties of human osteoarthritis subchondral bone osteoblasts: a basic science study. Arthritis Res Ther.

[3] Lajeunesse D, Massicotte F, Pelletier J, Martel-Pelletier J (2003). Subchondral bone sclerosis in osteoarthritis: not just an innocent bystander. Mod Rheumatol.

[4] Thysen S, Luyten F, Lories R (2015). Targets, models and challenges in osteoarthritis research. Dis Model Mech.

[5] Vane J, Botting R (1998). Anti-inflammatory drugs and their mechanism of action. Inflamm Res.

[6] Youssef P, Cormack J, Evill C, Peter D, Roberts-Thomson P, Ahern M (1996). Neutrophil trafficking into inflamed joints in patients with rheumatoid arthritis, and the effects of methylprednisolone. Arthritis Rheum.

[7] Reviews CT. e-Study Guide for: Slatter's Fundamentals of Veterinary Ophthalmology: Veterinary medicine, Veterinary medicine, 4th edition edn. Ventura, CA, USA: Cram101 Textbook Reviews; 2014.

[8] Dvorak L, Cook J, Kreeger J, Kuroki K, Tomlinson J (2002). Effects of carprofen and dexamethasone on canine chondrocytes in a three-dimensional culture model of osteoarthritis. Am J Vet Res.

[9] Huebner KD, Shrive NG, Frank CB (2014). Dexamethasone inhibits inflammation and cartilage damage in a new model of post-traumatic osteoarthritis. J Orthop Res.

[10] Abou-Raya A, Abou-Raya S, Khadrawi T, Helmii M (2014). Effect of low-dose oral prednisolone on symptoms and systemic inflammation in older adults with moderate to severe knee osteoarthritis: a randomized placebo-controlled trial. J Rheumatol.

[11] Chrysis D, Zaman F, Chagin A, Takigawa M, Sävendahl L (2005). Dexamethasone induces apoptosis in proliferative chondrocytes through activation of caspases and suppression of the Akt-phosphatidylinositol 3'-kinase signaling pathway. Endocrinology.

[12] Farkas B, Kvell K, Czömpöly T, Illés T, Bárdos T (2010). Increased chondrocyte death after steroid and local anesthetic combination. Clin Orthop Relat Res.

[13] Fubini S, Todhunter R, Burton-Wurster N, Vernier-Singer M, MacLeod J (2001). Corticosteroids alter the differentiated phenotype of articular chondrocytes. J Orthop Res.

[14] Nakazawa F, Matsuno H, Yudoh K, Watanabe Y, Katayama R, Kimura T (2002). Corticosteroid treatment induces chondrocyte apoptosis in an experimental arthritis model and in chondrocyte cultures. Clin Exp Rheumatol.

[15] Nganvongpanit K, Boonsri B, Sripratak T, Markmee P (2013). Effects of one-time and two-time intra-articular injection of hyaluronic acid sodium salt after joint surgery in dogs. J Vet Sci.

[16] Sun S, Hsu C, Sun H, Chou Y, Li H, Wang J (2011). The effect of three weekly intra-articular injections of hyaluronate on pain, function, and balance in patients with unilateral ankle arthritis. J Bone Joint Surg Am.

[17] Canapp S, Cross A, Brown M, Lewis D, Hernandez J, Merritt K (2005). Examination of synovial fluid and serum following intravenous injections of hyaluronan for the treatment of osteoarthritis in dogs. Vet Comp Orthop Traumatol.

[18] Moreland L-W (2003). Intra-articular hyaluronan (hyaluronic acid) and hylans for the treatment of osteoarthritis: mechanisms of action. Arthritis Res Ther.

[19] Rydell N, Balazs E (1971). Effect of intra-articular injection of hyaluronic acid on the clinical symptoms of osteoarthritis and on granulation tissue formation. Clin Orthop Relat Res.

[20] Takahashi K, Hashimoto S, Kubo T, Hirasawa Y, Lotz M, Amiel D (2001). Hyaluronan suppressed nitric oxide production in the meniscus and synovium of rabbit osteoarthritis model. J Orthop Res.

[21] Chaiwongsa R, Ongchai S, Tangyuenyong S, Kongtawelert P, Panthong A, Reutrakul R (2012). Chondroprotective potential of bioactive compounds of Zingiber cassumunar Roxb. against cytokine-induced cartilage degradation in explant culture. J Med Plants Res.

[22] Chotjumlong P, Khongkhunthian S, Ongchai S, Reutrakul V, Krisanaprakornkit S (2010). Human beta-defensin-3 up-regulates cyclooxygenase-2 expression and prostaglandin E2 synthesis in human gingival fibroblasts. J Periodontal Res.

[23] Siengdee P, Nganvongpanit K, Pothacharoen P, Chomdej S, Mekchay S, Ongchai S (2010). Effects of bromelain on cellular characteristics and expression of selected genes in canine in vitro chondrocyte culture. Veterinarni Medicina.

[24] Pradit W, Chomdej S, Nganvongpanit K, Ongchai S: Chondroprotective potential of Phyllanthus amarus Schum. & Thonn. in experimentally induced cartilage degradation in the explants culture model. In Vitro Cell Dev Biol Anim 2014, [Epub ahead of print]. 10.1007/s11626-014-9846-y 25515248

[25] Cesaretti M, Luppi E, Maccari F, Volpi N (2003). A 96-well assay for uronic acid carbazole reaction. Carbohydr Polym.

[26] Cook JL, Kuroki K, Visco D, Pelletier JP, Schulz L, Lafeber FP (2010). The OARSI histopathology initiative - recommendations for histological assessments of osteoarthritis in the dog. Osteoarthritis Cartilage.

[27] Brown C, Crawford R, Oloyede A (2007). Indentation stiffness does not discriminate between normal and degraded articular cartilage. Clin Biomech (Bristol, Avon).

[28] Király K, Lapveteläinen T, Arokoski J, Törrönen K, Módis L, Kiviranta I (1996). Application of selected cationic dyes for the semiquantitative estimation of glycosaminoglycans in histological sections of articular cartilage by microspectrophotometry. Histochem J.

[29] Milner PI, Wilkins RJ, Gibson JS, Rothschild DBM (2012). Cellular Physiology of Articular Cartilage in Health and Disease. Principles of Osteoarthritis- Its Definition, Character, Derivation and Modality-Related Recognition.

[30] Fadale P, Wiggins M (1994). Corticosteroid Injections: Their Use and Abuse. J Am Acad Orthop Surg.

[31] Fibel KH, Hillstrom HJ, Halpern BC (2015). State-of-the-Art management of knee osteoarthritis. World J Clin Cases.

[32] Ayhan E, Kesmezacar H, Akgun I (2014). Intraarticular injections (corticosteroid, hyaluronic acid, platelet rich plasma) for the knee osteoarthritis. World J Orthop.

[33] Davis D, Cyriac M, Ge D, You Z, Savoie F (2010). In vitro cytotoxic effects of benzalkonium chloride in corticosteroid injection suspension. J Bone Joint Surg Am.

[34] Bannuru R, Natov N, Obadan I, Price L, Schmid C, McAlindon T (2009). Therapeutic trajectory of hyaluronic acid versus corticosteroids in the treatment of knee osteoarthritis: a systematic review and meta-analysis. Arthritis Rheum.

[35] Abate M, Salini V, Chen PQ (2012). Hyaluronic Acid in the Treatment of Osteoarthritis: What is New. Osteoarthritis - Diagnosis, Treatment and Surgery.

[36] George E (1998). Intra-articular hyaluronan treatment for osteoarthritis. Ann Rheum Dis.

[37] Ozturk C, Atamaz F, Hepguler S, Argin M, Arkun R (2006). The safety and efficacy of intraarticular hyaluronan with/without corticosteroid in knee osteoarthritis: 1-year, single-blind, randomized study. Rheumatol Int.

[38] Guerne P, Blanco F, Kaelin A, Desgeorges A, Lotz M (1995). Growth factor responsiveness of human articular chondrocytes in aging and development. Arthritis Rheum.

[39] Strehl R, Schumacher K, de Vries U, Minuth W (2002). Proliferating cells versus differentiated cells in tissue engineering. Tissue Eng.

[40] Euppayo T, Siengdee P, Buddhachat K, Pradit W, Viriyakhasem N, Chomdej S, Ongchai S, Harada Y, Nganvongpanit K: Effects of low molecular weight hyaluronan combined with carprofen on canine osteoarthritis articular chondrocytes and cartilage explants in vitro. In: In Vitro Cell Dev Biol Anim. vol. [Epub ahead of print]; 2015. 10.1007/s11626-015-9908-9 25982358

[41] Pallante A, Bae W, Chen A, Görtz S, Bugbee W, Sah R (2009). Chondrocyte viability is higher after prolonged storage at 37 degrees C than at 4 degrees C for osteochondral grafts. Am J Sports Med.

[42] Hossain MA, Park J, Choi SH, Kim G (2008). Dexamethasone induces apoptosis in proliferative canine tendon cells and chondrocytes. Vet Comp Orthop Traumatol.

[43] Tu Y, Xue H, Francis W, Davies A, Pallister I, Kanamarlapudi V (2013). Lactoferrin inhibits dexamethasone-induced chondrocyte impairment from osteoarthritic cartilage through up-regulation of extracellular signal-regulated kinase 1/2 and suppression of FASL, FAS, and Caspase 3. Biochem Biophys Res Commun.

[44] Buddhachat K, Chomdej S, Nganvongpanit K. Co-administration of hyaluronic acid with anaeshetics, steriods, and NSAIDs in human articular chondrocytes culture in vitro. In: The 39th World Small Animal Veterinary Association Congress: 16-19th September 2014. Cape Town: Kenes International; 2014.

[45] Moo E, Osman N, Pingguan-Murphy B (2011). The metabolic dynamics of cartilage explants over a long-term culture period. Clinics (Sao Paulo).

[46] Bian L, Lima E, Angione S, Ng K, Williams D, Xu D (2008). Mechanical and biochemical characterization of cartilage explants in serum-free culture. J Biomech.

[47] Kozaci LD, Buttle DJ, Hollander AP (1997). Degradation of type II collagen, but not proteoglycan, correlates with matrix metalloproteinase activity in cartilage explant cultures. Arthritis Rheum.

[48] Lotz M, Otsuki S, Grogan S, Sah R, Terkeltaub R, D’Lima D (2010). Cartilage cell clusters. Arthritis Rheum.

[49] Ghosh P, Guidolin D (2002). Potential mechanism of action of intra-articular hyaluronan therapy in osteoarthritis: are the effects molecular weight dependent?. Semin Arthritis Rheum.

[50] Kawasaki K, Ochi M, Uchio Y, Adachi N, Matsusaki M (1999). Hyaluronic acid enhances proliferation and chondroitin sulfate synthesis in cultured chondrocytes embedded in collagen gels. J Cell Physiol.

[51] Lisignoli G, Grassi F, Zini N, Toneguzzi S, Piacentini A, Guidolin D (2001). Anti-Fas-induced apoptosis in chondrocytes reduced by hyaluronan: evidence for CD44 and CD54 (intercellular adhesion molecule 1) invovement. Arthritis Rheum.

[52] Responte D, Natoli R, Athanasiou K (2012). Identification of potential biophysical and molecular signalling mechanisms underlying hyaluronic acid enhancement of cartilage formation. J R Soc Interface.

[53] Gerwin N, Hops C, Lucke A (2006). Intraarticular drug delivery in osteoarthritis. Adv Drug Deliv Rev.

[54] Cyphert JM, Trempus CS, Garantziotis S: Size matters: molecular weight specificity of hyaluronan effects in cell biology. Int J Cell Biol. 2015, in press. (Article ID 563818). 10.1155/2015/563818 PMC4581549 26448754

[55] Craig E, Parker P, Camenisch T (2009). Size-dependent regulation of Snail2 by hyaluronan: Its role in cellular invasion. Glycobiology.

[56] Bourguignon L, Wong G, Earle C, Xia W (2011). Interaction of low molecular weight hyaluronan with CD44 and toll-like receptors promotes the actin filament-associated protein 110-actin binding and MyD88-NFκB signaling leading to proinflammatory cytokine/chemokine production and breast tumor invasion. Cytoskeleton (Hoboken).

[57] Lu Y, Evans C, Grodzinsky A (2011). Effects of short-term glucocorticoid treatment on changes in cartilage matrix degradation and chondrocyte gene expression induced by mechanical injury and inflammatory cytokines. Arthritis Res Ther.

[58] Jafari H, Sáez-Llorens X, Paris M, Rinderknecht S, Friedland I, Ehrett S (1993). Dexamethasone attenuation of cytokine-mediated articular cartilage degradation in experimental lapine Haemophilus arthritis. J Infect Dis.

[59] Florine E, Miller R, Porter R, Evans C, Kurz B, Grodzinsky A (2013). Effects of Dexamethasone on Mesenchymal Stromal Cell Chondrogenesis and Aggrecanase Activity: Comparison of Agarose and Self-Assembling Peptide Scaffolds. Cartilage.

